# How unmet are unmet needs post-stroke? A policy analysis of the six-month review

**DOI:** 10.1186/s12913-019-4210-2

**Published:** 2019-07-12

**Authors:** Vanessa Abrahamson, Patricia M. Wilson

**Affiliations:** Centre for Health Services Studies, University of Kent, Canterbury, CT2 7NX UK

**Keywords:** Stroke rehabilitation, Six-month review, Health and social care, Self-management

## Abstract

**Background:**

Stroke is the fourth largest cause of death in the UK and a leading cause of death and disability worldwide. Policy recommends reviewing patients at six-months post-stroke to identify unmet needs but lacks evidence of effectiveness. This study explored needs identified by patients, how they were addressed by the six-month review (6MR) and whether or not policy aspirations for the review were substantiated by the data.

**Methods:**

A multiple case study design underpinned by critical realism. Data sources included interviews with 46 patients and 28 professionals across three sites in the South East Coast of England. Patients’ interviews coincided with their reviews of which twenty-nine were observed. Thematic analysis of interviews, observations and policy documents was carried out within and across sites.

**Results:**

There were ‘hotspots’ in the care pathway where patients and carers felt particularly unsupported. Whilst these gaps exacerbated anxiety, they were neither universal nor ameliorated by review. Patients consistently identified unmet needs related to rehabilitation, information/education and support. Stroke nurse specialists focused on investigations, medication and liaising with general practitioners or consultants while the Stroke Association co-ordinator focused on sign-posting to other services and provision of generic information which not all respondents found helpful. The remit of review was more modest than that of policy aspirations.

**Conclusions:**

The review rests on two causal assumptions: that identifying unmet need will lead to its amelioration; and that provision of information will lead to behaviour change and self-management. While there was some evidence to support the former, there was almost none for the latter. The 6MR would benefit from a patient-led approach to its timing and format; a consistent and individualised approach to stroke education and self-management that is embedded across the care pathway; and targeting reviews should be considered.

## Background

There are more than 100,000 strokes in the UK each year and nearly a quarter will be followed by another stroke within five years [1]. The National Stroke Strategy [2] set out an ambitious ten-year framework that recommended reviewing all patients in England, Wales and Northern Ireland at six weeks, six-months and annually thereafter. Subsequent guidelines [3, 4] have endorsed this blanket approach but access remains limited [4].

Three key studies have highlighted unmet needs post-stroke in the UK. McKevitt et al. [5] estimated the prevalence of self-reported need amongst community dwelling adults in the UK (*n* = 1251), one to five years post-stroke. Half (51%) reported none and amongst the remainder the median was three (range 1–13): 54% reported a need for stroke information; 52% reduction or loss of work; and 18% loss of income. Secondly, the Care Quality Commission [6] identified significant shortcomings in services with many gaps post-discharge. Thirdly, the Stroke Association (SA) [7], a national organisation which lobbies on behalf of stroke survivors, reported that patients felt abandoned post-discharge, with needs including access to therapies, support, information and integrated working across health and social care.

The eligible population to receive a six-month review (6MR) is approximately 60,000 patients per year [8]. The only comprehensive audit of 6MR provision in England, albeit with a low response rate (36%), found that 6MR services were operational in just under one-third of clinical commissioning groups [9]. Similarly, the only general practitioner (GP) survey (*n* = 300) found much variation in provision, format and outcomes [10]. One-third of GPs were aware of the recommendations and just over half provided regular reviews but the focus was mostly limited to medical management [10].

### Evidence of effectiveness of the 6MR

To-date there is no evidence that the 6MR enhances recovery or leads to any statistically significant improvements. Most recently, Forster et al.’s [11] trial involved stroke care co-ordinators contacting patients at six and twelve months, using structured questions to assess need and develop a care plan. Even so, there were no statistically significant differences for any of the outcomes. Preceding this, Forster et al. [12] evaluated a structured reassessment of need at six months and again found no clinically significant benefits at twelve months although the intervention group did express greater satisfaction with information provision, the most common action.

Over ten years preceding the introduction of 6MRs, Forster and Young [13] evaluated an intervention consisting of nurse specialist visits over one year post-stroke, with a minimum of six visits in the first six months. Only patients with mild disability demonstrated small gains in social outcomes. Finally, a Cochrane meta-analysis evaluated the impact of a healthcare worker or volunteer whose roles were grouped under the title of ‘stroke liaison worker’ [14]. Sixteen trials were included but the review found no evidence of effectiveness with any of the interventions.

While there is no evidence of effectiveness, there is evidence suggesting that an individualised approach might be preferable [5, 11]. Given the heterogeneity of stroke patients this is unsurprising and we found that responses to review depended on contextual factors, presented elsewhere [15].

### Patient perspective on unmet need post-stroke

There have been few studies in England specifically exploring the patient perspective on unmet need one-year post-stroke and none appertaining to the 6MR. Shannon, Forster and Hawkins [16] interviewed a subset of respondents from the evaluation of a tool developed to carry out 6MRs; while clinicians identified a need as unmet, patients did not if they thought that further intervention would not ameliorate the need. Similarly, Sumathipala [17] interviewed 35 patients and highlighted a range of factors which affected how needs were perceived. Respondents self-reported impairments but most circumvented them by mobilising support from family and friends.

There have been few robust studies identifying carers’ unmet needs in the last decade [18] and although this paper does not focus on carers, it is worth noting that there does appear to be an association between the number of reported unmet needs and increasing carer burden [19, 20].

This paper addresses a gap in the literature relating to the review process. It focuses on patients’ perspective on unmet need post-stroke; how these needs were addressed by the 6MR; and whether or not policy aspirations for the review were substantiated by the data. Further findings from this study relating to the purpose, outcomes and underlying mechanisms of review are published elsewhere [15].

## Methods

A multiple case study design [21] underpinned by critical realism [22] was used. A case study approach allows in-depth study of a complex intervention in the exact socio-political context in which it is carried out [23] through exploring multiple perspectives in order to explain the rationale for what is under investigation. Yin’s case study approach is ‘orientated toward a realist perspective’ [21] and the two paradigms are well matched given the latter’s belief that there is an independent reality ‘out there’ but our knowledge of that reality is socially constructed [24].

In choosing multiple cases it was important to treat them as if they were multiple experiments using replication logic rather than sampling logic [21]. Each case consisted of a ‘whole’ study which was analysed separately alongside cross case analysis [21]. Three sites in the South East Coast of England were selected, based on their model of review, set within the context of local policies and demographics and were thus of theoretical interest. However, it was anticipated that the results would be similar in key aspects (a literal replication) because all services were, and still are, based on the same local guidance [25].

The focus, and unit of analysis, was the 6MR when stroke services have largely withdrawn and patients and carers have reported feeling abandoned by statutory services [7]. Patients were tracked from discharge home for up to one year, where possible, to include their annual review. The focus was the 6MR because six-week reviews occur when most patients are still receiving rehabilitation and annual reviews are largely unavailable.

Stroke nurse specialists (SNSs) provided 6MRs in sites 1–2. A SA co-ordinator, employed by the Stroke Association, provided reviews in site 3. Patients and carers were interviewed at about six weeks post-discharge, after their 6MR and where possible after their annual review. Providers of the review, therapists, managers, GPs and commissioners were also interviewed.

Ethical approval was granted by the Research Ethics Committee London-Surrey Borders (reference 15/LO/0808).

Although case studies refer to replication logic rather than sample size [21], based on admissions data for each site, it was estimated that a total of thirty patients would be feasible. For commissioners, reviewers and other clinicians, there were limited numbers of relevant individuals who were all approached. Exact numbers were informed by Hennink et al.’s [26] definition of *meaning saturation* or ‘the point when … no further dimensions, nuances, or insights of issues can be found’. Thus, exact numbers were determined by this view of saturation and paralleled normal practice within qualitative research [27].

Forty-six patients were interviewed (2015–16); twenty-four were followed for 6–12 months, fifteen for 12–18 months, and seven for less than 6 months. Twenty-eight professionals were interviewed and twenty-nine reviews observed. Table 1 summarises characteristics per site.

**Table 1:** Setting, approach and respondents per site

	Case study 1	Case study 2	Case study 3
Setting and model of review			
Who carried out the 6MR?	SNSs based in NHS Trusts that provided community stroke rehabilitation.	SNS employed by a social enterprise that provided community stroke rehabilitation. The SNS’s post was split between 6MRs and Early Supported Discharge.	SA co-ordinator.
Model of review	Medical model focused on medication, investigations and referrals to other statutory services.	Medical model supplemented by two sessions of a ‘life after stroke’ group which focused on self-management and a ‘personal stroke plan’ booklet provided in the acute setting.	Social model focused on sign-posting to community services such as exercise classes.
Timing of the 6MR	Generated by administrative system, mostly at 6 months.	Generated by administrative system, mostly at 6 months.	SA co-ordinator liaised with the community stroke team so reviews coincided with therapy withdrawing.
Were 6 week reviews provided?	Yes, by SNS and/or the stroke consultant^a^.	Yes, by the SNS in the acute setting and/or the stroke consultant^a^.	Some initial visits when requested by therapists and/or consultant reviews^a^.
Were annual reviews funded?	Yes	No	No
Respondents: patients			
Number of patients (and carers) interviewed after 6MR^b^	26 (14)	15 (11)	5 (5)
Patients’ age range, years.	28-88	31-91	67-80
Number of patients of working age at time of stroke	11	6	1
Number of patients with other long-term conditions	13	6	3
Number of patients who were married or co-habiting	17	10	4
Respondents: reviewers & others			
Stroke nurse specialists	3	3	-
SA co-ordinator	-	-	1
SA support workers	1	1	-
Physio/ occupational therapist, service manager, GP or commissioner	6	5	2
Across site interviews including SA managers	6		
Observations of reviews			
6MR	10	9	4
1 year review	6	-	-

^a^Consultant reviews were medical rather than a structured 6MR; some consultants liaised with SNSs to avoid duplication

^b^Patients/carers were interviewed to coincide with their 6 week, 6 month, and (where possible) annual review

Data consisted of semi-structured interviews, observations and policy analysis. All interviews were digitally recorded and transcribed verbatim, with consent. Observations were structured using Lofland’s [28] framework and included reviews, team meetings and a ‘life after stroke’ group. Observations of reviews provided a different but valid perspective to interviews [29] and were compared against policy guidelines as part of triangulating the data. All data were imported into Nvivo to create a transparent and organised database.

Analysis of interviews, observations and policy documents drew on thematic analysis [30] and was informed by Yin’s [21] approach. After familiarisation with the data, a combination of mind maps, annotations and memos were used across all sources to generate initial descriptive codes; original wording was retained to aid rigor. Second level coding involved a similar process but in relation to the whole data set to consider whether the codes accurately reflected the whole dataset. Integrating the dataset involved looking for relationships and conceptual categories across sites with multiple sources of data and multiple perspectives, in-line with both critical realism and case study approach. Patient (and carer) views about the 6MR were cross-referenced with what the reviewer had commented, observations and policy documents to seek further evidence to understand underlying mechanisms and adjust analysis accordingly. Theory building used explanation building and logic models with the goal of explaining the phenomena by identifying the underlying mechanisms [21].

Rigor, or trustworthiness, was addressed through the criterion of credibility, transferability, dependability and confirmability [31]. A combination of approaches included prolonged engagement with the data and thick description [31]; multiple sources of data [32]; independent coding of transcripts by both authors [21, 30]; negative case analysis [21]; establishing a chain of evidence and maintaining an audit trail [21]; and reflexivity to maintaining vigilance for researcher bias [33].

## Results

This section focuses on patients’ perceived needs and how reviewers attempted to ameliorate them; the data is compared to policy aspirations. Patients consistently identified needs related to rehabilitation, education/information and support.

### Perceived needs for community stroke rehabilitation

There were ‘hotspots’ where some patients and carers felt unsupported. These occurred during transitions between units, discharge home, waiting for community rehabilitation to commence and when services withdrew. In site 3, the SA co-ordinator timed reviews to coincide with therapy withdrawing but in other sites this was not the case. Whilst these gaps exacerbated respondents’ anxiety, they were neither universal nor ameliorated by review. A few respondents reported feeling abandoned but more commonly respondents simply wanted to progress and were aggrieved by delays in community rehabilitation starting and not being kept informed:

*It’s a long time to wait before they came round, I wanted to get moving because the physio was so good in hospital … but then when you come home there’s nothing … I wanted to just get going and build on what I was doing in the hospital* (Site 3, female, 79 yrs., interview)

Many respondents wanted more intensity, frequency and duration of therapy and perceived this as an unmet need which the 6MR did not address. For example, this self-employed respondent stated:

*We had originally been promised [community therapy] twice a week and it was only once a week and then we had to complain and eventually they said, “Oh right, okay, we’ll do twice a week”* (Site 2, male, 63 yrs., interview)

In some cases it was possible to verify delays and limited input, which therapists attributed to staffing but in other cases it appeared related to mismatched expectations. Respondents had a longer-term outlook and wanted to continue rehabilitation even when progress had plateaued. However, therapists had to withdraw services when they could no longer identify achievable goals, in-line with policy [4]. The rationale was that patients needed to move onto community facilities, caseloads were large and it was part of adapting to long-term disability:

*It was exactly around this whole adjustment issue … even though he has done very well in our eyes, he is not back to normal, and they just want therapy for ever* (Site 2, community therapist, interview)

Younger respondents expressed needs specific to their situation, mainly return to work and financial. However, they were not ready to resume work until well after rehabilitation had finished and the 6MR did not address this:

*The needs for people who are my age are a lot different … We need to be able to try and get back at it [work] as soon as we can* (Site 1, male, 28 yrs., interview)

Differences in perceptions of what therapy could achieve left a gap where patients did not feel equipped to manage everyday life but had no ongoing support and their 6MR was not due for several months.

### Perceived need for information, education and support

The need for ongoing support linked with the need for education along the care pathway. While information was provided during the inpatient phase, respondents expressed difficulty absorbing it because they felt overwhelmed, staff were rushed and the environment chaotic.

Alongside feeling overwhelmed, a few respondents did not know what to ask because they had no prior knowledge or experience:

*The doctors always said … please feel free to ring up … But it’s like all these things, you don’t know the questions to ask. You’ve no idea.* (Site 1, female, 63 yrs., interview)

In site 3, the SA co-ordinator provided standard information packs which most respondents had already received as inpatients but some found of limited relevance:

*I [researcher] noticed Stroke Association pack in a plastic bag behind sofa so asked if he had read it - said it was too generic, not relevant to his age* (Site 2, male, 31 yrs., observation fieldnotes)

When asked what information respondents would have liked, or liked more of, there were two main areas. Firstly, what was going to happen in terms of their immediate treatment, transfers to other units and discharge home. Secondly, many respondents wanted more information about aetiology, prognosis and secondary prevention, as policy recommends [4]. During observations of reviews, respondents frequently asked about diet, exercise and fatigue which remained unaddressed for the months preceding their review:

*That [information] was fairly zero, actually! I would have liked more information about how to prevent another stroke and also … any alarm signals* (Site 2, female, 76 yrs., interview)

Some respondents had follow-up appointments with the stroke consultant but sometimes the appointment felt too rushed to ask questions whereas the 6MR allowed respondents time to reflect and clarify information.

In site 2, respondents were invited to a ‘life after stroke’ group which consisted of two sessions covering secondary prevention and self-management. The timing of the group in relation to their 6MR varied but from observations, it appeared to act as an additional point at which respondents could seek reassurance. Most appreciated was advice from the dietician and one-to-one time afterwards with the SNS:

*Dietician started with quiz to engage people; she focused on what we should eat rather than what we shouldn’t; had boxes of packaging to look at [food] labels; lots of questions asked; someone said how helpful it was to talk to SNS afterwards* (Site 2, Life After Stroke group, observation fieldnotes)

This site also used a ‘personal stroke plan’ which was a booklet that patients received on admission to the stroke unit and included staff names, therapy goals and general information. However, few patients found it helpful, the majority had not been completed and most were not used in the community, as intended.

Many people used the internet to find out about stroke, re-training packages, clinical trials, equipment and secondary prevention. Some resorted to the internet because they felt the information they had received was insufficient, or too medicalised, while others wanted to supplement what they had been told:

*I try to acquire knowledge elsewhere, so I take what they [therapists] tell me and what I learn online as well and use it* (Site 1, female, 37 yrs., interview)

### Unmet needs addressed by the 6MR

We have previously reported how 6MRs carried out by SNSs were found to be more medically orientated than those completed by a Stroke Association co-ordinator who focused on social issues [15]. This section expands on what needs were identified and how they were addressed, compared to policy guidelines [4, 25].

#### Medical follow-up

SNSs focused on investigations, medication and liaising with GPs or consultants. They looked for medical issues that might have been overlooked, for example undiagnosed atrial fibrillation or sleep apnoea, and followed up as necessary:

*It is fairly typical that when I see [consultant] I have several heart monitors for him to order and I generally would ask the GP to prescribe the right meds, but I back it up with a letter from [consultant]. The junior doctors complete the EDNs [electronic discharge notifications] and order the investigations and sometimes there are oversights* (Site 1, SNS, interview)

The SNS was rectifying oversights so whether this equated with unidentified need, as defined by policy [4], is arguable. Similarly, when patients highlighted delays in follow-up appointments SNSs were able to chase directly. Many respondents found this medical focus on secondary prevention reassuring.

#### Referrals to statutory services

SNSs were able to directly refer patients to services within the NHS, such as falls clinics, while the SA co-ordinator advised patients to ask their GP which in some cases had already proved unproductive. However, some services were unavailable or had a long waiting list, particularly neuropsychology. Reviewers seldom referred patients for further rehabilitation or asked about therapy (or personal) goals despite recommendations that rehabilitation goals should be reviewed [4]. Other therapy needs that would have benefited from review included positioning, seating, muscle tone and splinting. For example:

*She was sitting badly in wheelchair, internal rotation hips, saggy canvas, no cushion, no armrest for left arm, footrests need adjusting. Wearing splint for left leg but looks too tight … Wearing arm sling incorrectly (as when interviewed), arm hanging from subluxed shoulder* (Site 1, female, 63 yrs., observation fieldnotes)

Guidelines state that therapy can only be offered ‘if goals for specific functions and activities can be identified and agreed and the potential for change is likely’ [4] but this is from the perspective of clinician, not patient. However, none of the reviewers felt equipped to address therapy issues and raised concerns about inappropriate re-referrals:

*Sometimes there are those patients who I think ‘would you benefit from physio again?’ … I don’t want to overload the already limited service with people, I do wonder sometimes am I a little bit over cautious* (Site 2, SNS, interview)

In contrast, one manager, previously a therapist, regarded reviewing goals as an intrinsic part of the process:

*For me, it would be revisiting all the goals that you originally had and hopes and desires to see if any of those have come to fruition; to see if they haven’t why they haven’t, and to see if … re-referral back into any of the rehab systems would be of benefit* (Site 2, manager, interview)

Ongoing vestibular, visual and visuo-perceptual disorders appeared to be another unresolved need that respondents felt had not been adequately addressed prior to review. Despite acknowledging these symptoms, respondents were not always referred to appropriate specialists.

#### Signposting and provision of information

Reviews carried out by the SA co-ordinator focused on signposting respondents to community facilities and groups to which the patient could self-refer. The co-ordinator had excellent knowledge:

*Our role is about signposting, we may make referrals to certain services but it’s very much about signposting, giving information because of your time restrictions* (Site 3, SA co-ordinator, interview)

The SNSs also signposted respondents to other services, often the SA, but this was less prominent and related more to medical appointments:

*I’m a sign-poster. I make sure patients get the right service through referring them to the right people* (Site 1, SNS, interview)

Although fieldnotes commonly recorded reviewers referring or signposting to other services, reviewers did not have any mechanism to check the outcome. If time allowed, they would follow-up, and the SA co-ordinator had more leeway to do so, but this was not always possible.

#### Secondary prevention and self-management

It was observed during reviews that SNSs provided tailored information related to medical aspects of secondary prevention while the SA co-ordinator concentrated on lifestyle factors. Both aimed to encourage self-management. Many respondents were aware of generic health promotion messages related to eating, exercise, alcohol and smoking so the review appeared to endorse prior knowledge, for example:

*Reviewer advised ‘everything in moderation … olive oil rather than animal fat … the other key thing is fruit and veg’. Wife stated she didn’t fry [food], they eat salad and veg. She’s diabetic so watches her diet. Aware of warfarin contraindications* (Site 3, male, 73 yrs., observation fieldnotes)

A few respondents wanted to change their behaviour but lacked motivation and the 6MR prompted them to do so. For example, one respondent had been drinking heavily and the review prompted him to reduce his intake. His wife had dementia:

*I’ve known for a long that I was drinking too much and I was using it as a crutch because of the worry about my wife* (Site 1, male, 77 yrs., interview)

Many respondents were already effectively self-managing their condition, for example, monitoring their blood pressure and regulating their diet while others were more questioning or rejected the advice. Reviewers were realistic about how much they could achieve and acknowledged limitations:

*They all tell me they’re on a healthy diet but I don’t believe it … they’ll be overweight or … they’re diabetic and they’ve got chocolate on the side … you get to know whether they’re interested or not* (Site 1, SNS, interview)

Many respondents wanted to increase their exercise levels but struggled to do so, especially those with severe hemiparesis. Although reviewers were observed providing information on exercise classes there were gaps in services and difficulty with access that precluded the most disabled.

Only one commissioner questioned what a single review could achieve and suggested that self-management needed to be supplemented, for example with an online intervention, because this aspect was not sufficiently embedded into the review:

*Asking people questions about their diet, their exercise habits, their drinking habits, smoking habits … is supporting someone’s self-management, but that … is only a brief intervention. I think that you would need longer-term support and perhaps quite specific coaching* (Site 1, manager, interview)

Although reviewers provided a ‘structured health and social care review’ [4], the remit was more modest than locally defined policy aspirations [25] based on these guidelines, or those of the Stroke Association [34], as Table 2 summarises.

**Table 2:** What evidence supported outcomes for the 6MR?

Locally defined patient outcomes based on national guidelines	Any evidence from the data
Greater patient involvement in identifying and planning to address their ongoing needs.	Minimal evidence, and only for those who were already pro-active in addressing their ongoing needs.
Access to a wide range of information about NHS, voluntary, community and social services that will contribute to achieving stroke related goals.	The SA co-ordinator provided comprehensive information about local services; the SNSs provided limited information and/or advised the patient to contact the SA.
Feeling supported and more confident.	Limited evidence, mainly those who were confident and had good social support.
Will be less likely to be readmitted to hospital.	No evidence but SNSs did identify medical concerns requiring follow-up (and urgently, in a few instances).
Will be less likely to have another stroke.	As above.
Improved health and general well-being.	No evidence but indirectly the review may have contributed to some improvement for those who were more able and articulate.
Reduced GP appointments.	No evidence.
Reduced dependency on social services.	No evidence.
Stroke Association overall service outcomes	
Improved quality of life	Potentially an indirect outcome through signposting respondents to community services.
Improved medication compliance	No evidence for SA reviews but those reviewed by SNSs valued their tailored medical advice and expertise which could have led to improved adherence.
Reduced hospital admissions	No evidence.
Reduced social isolation	As for improved quality of life – potentially an indirect outcome.

## Discussion

The results explored the perspective of patients and reviewers and compared these with policy aspirations. The case study approach, combined with a realist perspective, enabled exploration of underlying mechanisms and contextual issues. However, policy does not acknowledge the importance of context instead adhering to a rigid timeframe and uniform approach [4], an exemplar of a blanket approach despite no evidence to support it [11–13].

McKevitt et al. [5] suggested a targeted approach for stroke patients given that half of those surveyed did not report unmet needs and more recently Forster et al. [11] suggested that a ‘targeted more bespoke intervention’ might be preferable. Andrew et al. (2015) also emphasised that effective interventions should be personalised and responsive over time and recommended ‘regular review and a point of contact for trouble-shooting and reassessment when situations change’ [19], similar to the 6MR but patient-led.

One way of understanding the disconnect between recipients and providers is to consider the 6MR as a complex intervention introduced into complex social situations [35]. Although Pawson (2013) attempts to distinguish realist evaluation from critical realism, the differences are not as significant as Pawson contends [36] which allows us to draw on his understanding of context alongside Bhaskar’s stronger representation of agency [36]. Pawson [35] identifies four contextual layers all of which resonated with the 6MR: i) *individuals*, or the characteristics and capacities of stakeholders, as evidenced by respondents’ interpretation of need compared to that of reviewers’ medical or social orientation; ii) *interpersonal relations* between reviewers and patients which was, to a certain extent, pre-determined by preceding events [15]; iii) *institutional settings* or the rules, norms and customs local to the 6MR which constrained reviewer’s agency; and iv) *infrastructure*, or the wider socio-economic climate, in particular resource limitations which restricted what could be offered to meet identified needs. These factors explain the complexity of the intervention and why a uniform approach to review is likely to be of limited benefit.

### Policy aspirations versus reality

The review policy was informed by limited evidence of unmet need and that some patients felt abandoned post-discharge [6, 7]. However, this was not a major finding in our study - more commonly, respondents felt unsupported at key ‘hotspots’ and frustrated with delays in therapy and/or its limited duration, again dictated by policy [4]. Despite reviewers’ patient-centred approach, and site 3’s emphasis on timing 6MRs to coincide with therapy withdrawing, there were areas that the 6MR did not adequately address, particularly related to therapy needs and goals. These omissions reflected barriers imposed by insufficient time and a rigid format not tailored to individual needs. To a certain extent, this was countered by reviewers’ personal attributes and professional expertise, both of which engendered trust and appeared to be the mechanism by which outcomes were achieved. However, policy expects reviewers to address all medical, social and emotional aspects of recovery for patient and carer in a one-off, time-limited intervention [4] in the context of limited statutory or voluntary services and long waiting lists.

The review policy rests on two causal assumptions: that identifying unmet need will lead to its amelioration; and that provision of information will lead to behaviour change, self-management and secondary prevention. While there was some evidence to support the former, there was almost none for the latter. However, this is hardly surprising given the 6MR’s parameters and the complexity of the recovery process.

It is challenging for a written policy to capture the complexity of individual responses post-stroke including the complexity of human behaviour, underlying mechanisms and the socio-economic context. The 6MR attempts to marry social and medical aspects without an underpinning theory. This limits its potential, and increases the likelihood of non-adherence, given that respondents will draw on their own illness understanding and bring these to the 6MR [15]. Similarly, while reviewers tried to incorporate self-management this was limited given that it is a complex intervention that needs to be individualised and consolidated over time [37].

Pindus et al. [38] carried out a systematic review (1996–2015) and meta-analysis of patients and carers experiences of primary care and community healthcare services post-stroke. Of the 51 studies, 16 were UK based and published after the National Stroke Strategy [2]. Needs were categorised into four areas: continuity of care and support from community services; the quality of communication; information provision; and limited access to services, especially rehabilitation and emotional support. These unmet needs all resonate with our study findings but were largely outside the remit of 6MRs.

The main study limitation was delays in gaining access to site 3 which significantly reduced the number of participants recruited and length of follow-up. We were unable to gain permission for a second Stroke Association site or one where therapists carried out reviews. We had to exclude patients discharged to care homes because of ethical concerns and capacity to consent.

## Conclusion

Policy aspirations for the 6MR were not substantiated and the ‘one size fits all’ approach constrained its potential. There have been no policy changes since data collection that might affect 6MR delivery. Our findings suggest that for the 6MR to have impact, it would be preferable to: i) instigate a patient-led approach to its timing and format; ii) embed the review process within rehabilitation so that there is a consistent approach to provision of information which is individualised and consolidated at each stage along the care pathway; iii) similarly, integrate self-management into the care pathway and re-visit it at each review within the context of multiple co-morbidities; iv) consider targeting the review [5, 11].

## Data Availability

The datasets used and/or analysed during the current study are available from the corresponding author on reasonable request.
